# Killing two birds with one stone: trans-kingdom suppression of PAMP/MAMP-induced immunity by T3E from enteropathogenic bacteria

**DOI:** 10.3389/fmicb.2014.00320

**Published:** 2014-07-22

**Authors:** Malou Fraiture, Frédéric Brunner

**Affiliations:** Plant Biochemistry, Centre for Plant Molecular Biology, Eberhard Karls University TübingenTübingen, Germany

**Keywords:** PAMP/MAMP, innate immunity, mammals, plants, enteropathogenic bacteria, *Salmonella*, *Escherichia coli*, type three effectors

## Abstract

Within the past decade, remarkable similarities between the molecular organization of animal and plant systems for non-self discrimination were revealed. Obvious parallels exist between the molecular structures of the receptors mediating the recognition of pathogen- or microbe-associated molecular patterns (PAMPs/MAMPs) with plant pattern recognition receptors strikingly resembling mammalian Toll-like receptors. Mitogen-activated protein kinase cascades, leading to the transcriptional activation of immunity-associated genes, illustrate the conservation of whole molecular building blocks of PAMP/MAMP-induced signaling. Enteropathogenic *Salmonella* and *Escherichia coli* use a type three secretion system (T3SS) to inject effector proteins into the mammalian host cell to subvert defense mechanisms and promote gut infection. Lately, disease occurrence was increasingly associated with bacteria-contaminated fruits and vegetables and common themes have emerged with regard to whether and how effectors target innate immune responses in a trans-kingdom manner. We propose that numerous *Salmonella* or *E. coli* effectors may be active *in planta* and tend to target central components (hubs) of immune signaling pathways.

## INTRODUCTION

Animals and plants are able to discriminate between self and non-self, which is the key feature to fight against microbial pathogens. The first line of the innate immune response in both animals and plants is induced by the perception of common pathogen or microbe-associated molecular patterns (PAMPs/MAMPs) that are absent from host cells (Nurnberger et al., 2004; Ausubel, 2005; Akira et al., 2006; Boller and Felix, 2009; Macho and Zipfel, 2014). PAMP/MAMP sensing is mediated by specific pattern recognition receptors (PRRs), localized in different sub-cellular compartments. In mammals, the transmembrane Toll-like receptor family (TLRs) and the cytoplasmic nucleotide-binding oligomerization domain (NOD)-like receptor family (NLRs) are the most representative PRRs (Kawai and Akira, 2011; Kumar et al., 2011). In plants, typical PRRs belong to the class of transmembrane receptor-like kinases or membrane bound- receptor like proteins with extracellular leucine-rich repeat- (LRR-RLKs/LRR-RLPs) or lysine motif- (LysM-RLKs/LysM-RLPs) containing domains (Boller and Felix, 2009; Macho and Zipfel, 2014). PAMP/MAMP binding induces intracellular signaling cascades leading to cellular re-programming. In mammals, the immune response is associated with the transcriptional activation of immunity-associated genes and production of cytokines including interleukins (ILs) or tumor necrosis factors (TNFs) and antimicrobial peptides. In plants, anti-microbial metabolites (phytoalexins) and proteins (Pathogenesis-Related proteins) are produced in response to infection. The ultimate outcome of the process, called inflammatory response in animals and PAMP/MAMP-triggered immunity (PTI/MTI) in plants, is pathogen clearance.

Successful pathogens have learned to subvert PAMP/MAMP triggered immune responses by producing effectors that contribute to virulence. Many effectors originated from human pathogenic enterobacteria, e.g. *Salmonella, shigella, Yersinia,* and *Escherichia coli* or plant pathogenic bacteria e.g. *Pseudomonads* and *Xanthomonads* were shown to manipulate host PTI/MTI signaling (Gohre and Robatzek, 2008; Brodsky and Medzhitov, 2009; Dean, 2011; Dou and Zhou, 2012; Johannessen et al., 2013; Raymond et al., 2013). Although enterobacteria do not represent a threat to agriculture, cumulative evidence support the view that, against the general dogma, *Salmonella* and some *E. coli* strains (O157:H7 serogroup) can actively invade, proliferate and spread in plants (Holden et al., 2009; Schikora et al., 2012). A prerequisite for *Salmonella* or *E. coli* growth on host plants would be the ability to cope with the immune system, notably through the evolution of a functional type three secretion system (T3SS) capable to breach through the cell wall and inject type three effectors (T3E) that manipulate immunity-associated components.

Here, we will review the parallels (and differences) between PAMP/MAMP-induced immune signaling in plants and animals with emphasis on the molecular components that are functionally conserved between both kingdoms. Further, we will discuss the potential of candidate effectors from animal- and plant-adapted enteropathogenic bacteria to suppress the evolutionary conserved PAMP/MAMP-triggered immune response.

## PAMP/MAMP-TRIGGERED IMMUNITY IS EVOLUTIONARY CONSERVED ACROSS KINGDOMS

**FIGURE 1:**
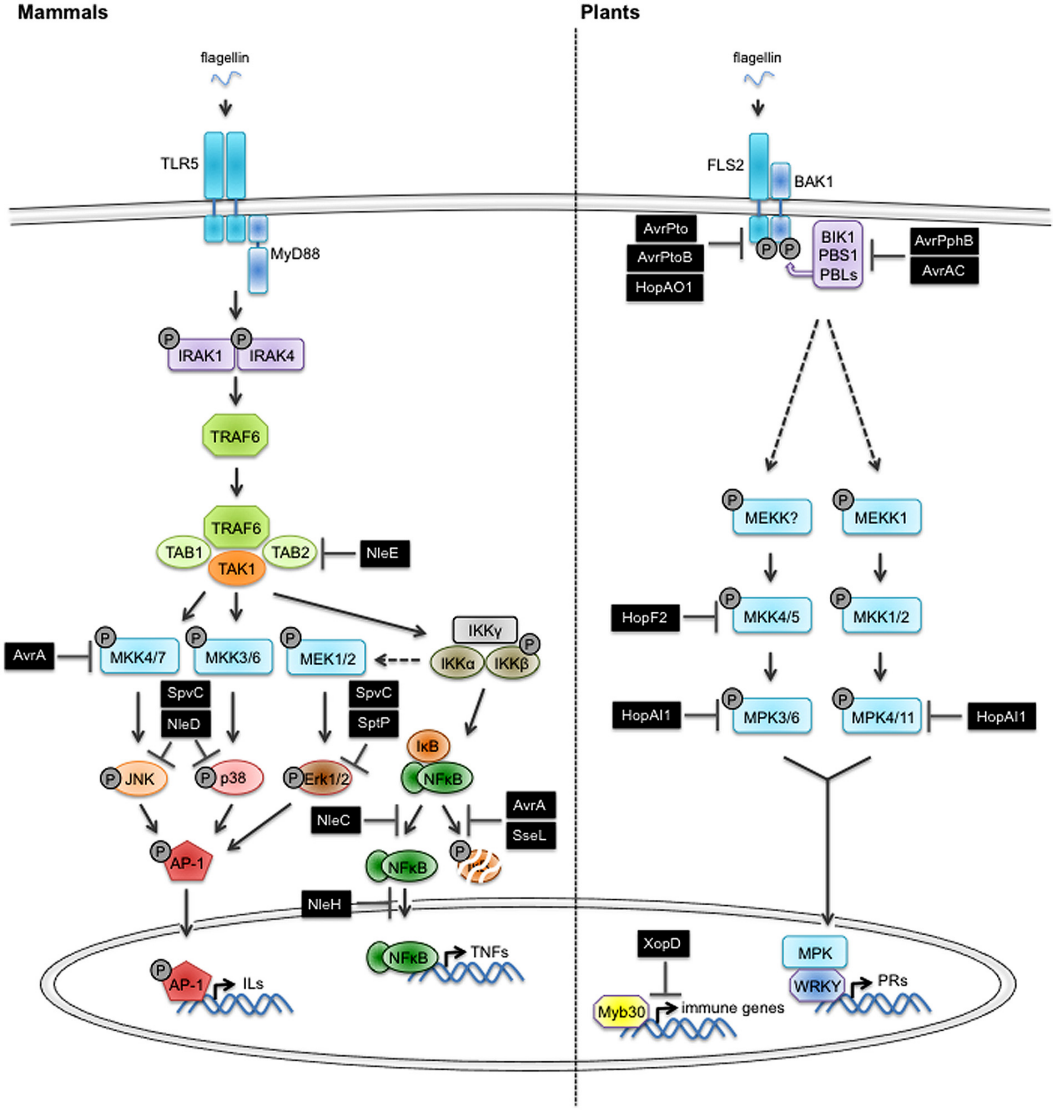
**Overview of the PTI/MTI-suppressing function of type three effectors from enterobacteria and phytopathogenic bacteria.** The perception of pathogen/microbe-associated molecular patterns (PAMPs/MAMPs) by pattern recognition receptors (PRRs) triggers signaling pathways conferring immunity (PTI/MTI) that are conserved in mammals and plants. Host-adapted bacterial pathogens use the type three secretion system (T3SS) to inject effectors (T3E) that compromise PTI/MTI. Components of basic plant defense and interfering pathogen effectors (black box) are depicted. See main text for additional details. Abbreviations used in the figure: TLR5, Toll-like receptor 5; FLS2, Flagellin Sensitive 2; MyD88, Myeloid Differentiation primary response gene 88; BAK1, BRI1-Associated receptor Kinase 1; BIK1, Botrytis-Induced Kinase 1; PBS1, AvrPphB susceptible 1; PBL, AvrPphB susceptible 1-like; IRAK1,4, Il-1 receptor associated kinase 1,4; TRAF6, TNF receptor associated factor 6; TAB1,2, TAK1 binding protein 1,2; TAK1, TGFβ activated Kinase 1; MEKKs, mitogen-activated protein kinase kinase kinases; MEKs/MKKs, mitogen-activated protein kinase kinases; MPKs, mitogen-activated protein kinases; JNK, c-Jun N-terminal kinase; p38, p38 mitogen-activated protein kinase; Erk, extracellular signal regulated kinase; IKKs, IkappaB kinases; IκB, inhibitor of kappa B; NF-κB, nuclear factor-kappa B; AP-1, activator protein 1; WRKY, WRKY transcription factor; MYB30, MYB domain protein 30.

The identification and functional characterization of PAMPs/MAMPs and their corresponding PRRs in mammals and plants underwent a burst of interest in the past two decades, contributing to drastically increase our fundamental knowledge about the molecular mechanisms of host adaptation to microbial infection. Best-studied PAMPs/MAMPs in mammals are lipopolysaccharides (LPS) from Gram- bacteria, peptidoglycan (PGN) from Gram+ bacteria, flagellin (the major constituent of bacterial flagella), double stranded RNA of viruses or bacterial DNA. In mammals, transmembrane TLRs and cytoplasmic NOD proteins with leucine rich repeats (LRR) are responsible for the recognition of PAMPs/MAMPs (Akira et al., 2006; Kawai and Akira, 2011; Kumar et al., 2011). The heterodimerization between different TLRs increases the potential of recognized PAMPs/MAMPs (Ozinsky et al., 2000). TLR5 is a good representative of the TLR family in mammals (**Figure 1**). It senses flagellin from a variety of different Gram+ and Gram- bacteria (Nempont et al., 2008) by recognizing the highly conserved N-terminal 99 amino acids and C-terminal 416-444 amino acids of the protein (Smith et al., 2003). Flagellin-induced signaling recruits adaptor proteins such as MyD88 (Myeloid Differentiation primary response gene 88). The adaptor then recruits IRAK1 and IRAK4 (IL-1R-associated kinases) causing their autophosphorylation and the association with TRAF6 (TNF-receptor-associated factor 6). TRAF6 activates the TAK complex (TAB1,2–TAK1 binding protein 1,2- and TAK1–TGFβ activated Kinase 1) which then induces the activation of MEK1/2, MKK3/6, 4, 7 (MAP kinase kinase 1/2, 3/6, 4, 7) and of the IKKγ(NEMO)/IKKα/IKKβ complex (IKK complex). The activated MKKs in turn activate ERK (Extracellular signal Regulated Kinase), JNK (c-Jun N-terminal Kinase), and p38 MAP kinase which ultimately induce the expression of immunity-associated genes like IL-1 (interleukin-1), IL-2, IL-6, and IL-12 through stimulation of the transcription factor AP-1 (Activator Protein 1). The activation of the IKK complex results in the degradation of I-κB (Inhibitor of kappaB) and the translocation of NF-κB (Nuclear Factor-kappaB) to the nucleus where it regulates the expression of genes encoding TNFs (Akira et al., 2006; O’Neill and Bowie, 2007; Kumar et al., 2011).

Most of the PAMPs/MAMPs, which are recognized in mammals, also trigger defense responses in plants. Like in animals, the perception is executed by cell surface receptors that are structurally very similar to TLRs. One of the best-studied PRR in plants is FLS2 (Flagellin Sensitive 2) from *Arabidopsis thaliana*, a protein with an extracellular leucine rich repeat domain (LRR) responsible for flagellin binding and an intracellular kinase domain involved in signal transduction (Gomez-Gomez and Boller, 2000; **Figure 1**). FLS2 homologs are present in nearly all the plant species that were tested for responsiveness to flagellin (Boller and Felix, 2009). FLS2 binds a conserved 22 amino acid peptide (flg22) located in the N-terminal part of flagellin. Flg22 binding leads to the recruitment of BAK1 (BRI1-Associated Kinase 1), another transmembrane receptor-like kinase, to form a functional receptor complex (Chinchilla et al., 2007; Heese et al., 2007; Sun et al., 2013). The FLS2-BAK1 complex undergoes several events of cross-phosphorylation at multiple serine/threonine and tyrosine residues that are located in the kinase domain (Schulze et al., 2010; Schwessinger et al., 2011; Yan et al., 2012). These phosphorylation events are thought to contribute to signaling initiation and specificity but their functions remain largely hypothetical (Macho and Zipfel, 2014). The receptor-like cytoplasmic kinase BIK1, constitutively associated with FLS2 in absence of flg22 also becomes phosphorylated and dissociates from the receptor complex (Lu et al., 2010; Zhang et al., 2010). The NADPH oxidase RbohD has been identified recently as a substrate of BIK1. Thus, a direct mechanistic link could be established between the formation of the receptor complex and the production of reactive-oxygen species (Kadota et al., 2014; Li et al., 2014). In contrast, the activation mechanism of MAP kinase cascades comprising MEKK1, MKK1/MKK2, MPK4 and MKK4/MKK5, MPK3/MPK6 downstream of the FLS2 receptor remains elusive (Asai et al., 2002). Activated MAP kinases are translocated to the nucleus where they regulate the expression of immunity-associated genes, for example by activating the transcription of genes encoding pathogenesis-related (PR) proteins.

FLS2 and TLR5 recognize different epitopes in flagellin. Therefore it is considered that animals and plants have developed their perception systems for flagellin independently. It is generally admitted that the similarity between the components present in the plant and animal innate immune signaling pathways is likely based on convergent evolution. Plants do not have downstream signaling components homologous to the MyD88 adaptor protein and intracellular TIRAP (Toll-interleukin receptor containing adaptor protein). The Rel family of transcription factors, which includes NF-κB in mammals, is also missing in plants. On the contrary, animals do not have transcription factors homologous to the WRKY family found in *Arabidopsis*.

## BACTERIAL TYPE THREE EFFECTORS MANIPULATING PAMP/MAMP-TRIGGERED IMMUNE SIGNALING COMPONENTS

Successful pathogens are able to manipulate PAMP/MAMP-triggered immunity in both animals and plants by producing effectors. Most of the Gram- bacteria use a sec-independent protein delivery system, the so-called type three secretion apparatus (T3SS), to translocate type three effectors (T3E) into the cytosol of host cells.

The molecular mechanisms underlying *Salmonella* and *E. coli* infection in humans are well-studied and the functions of T3Es become apparent. More than thirty T3Es have been identified in *Salmonella* and some of them have been shown to manipulate key cellular functions implicated in immunity (McGhie et al., 2009; Dean, 2011; Figueira and Holden, 2012). Many effectors are GTPase-activating proteins (GAPs) that interact and alter the function of the Rho family GTPases, proteins regulating numerous cellular processes including immune responses and cytoskeleton re-arrangements (Bokoch, 2005; Aktories, 2011). The *Salmonella* effector SptP contains a GAP domain and was shown to inhibit the downstream activity of Rac1 and Cdc42, two Rho family GTPases (Fu and Galan, 1999; Bruno et al., 2009). In addition, the C-terminal domain of SptP displays tyrosine phosphatase activity that blocks the MAP kinase pathway by inhibiting Raf kinase activation (Lin et al., 2003).

SpvC, a homolog of OspF from *Shigella flexneri*, is an effector with phosphothreonine lyase activity that exerts its inhibitory action on active MAP kinases and downregulates the expression of cytokines in infected cells (Mazurkiewicz et al., 2008). The MAP kinases are irreversibly inactivated through β-elimination of the phosphate group from a conserved phosphothreonine residue in the activation loop (Li et al., 2007; Zhu et al., 2007).

Another well-studied *Salmonella* effector, AvrA, is a close homolog of YopJ from *Yersinia spp*. It was shown that AvrA possesses acetyltransferase activity. Through its interaction with MKK4 and MKK7, it inhibits the activation of the JNK pathway without interfering with the activation of the NF-κB or p38 pathways (Jones et al., 2008). Interestingly, AvrA itself is activated through phosphorylation that is dependent on the stimulation of the extracellular signal-regulated kinases 1/2 (Erk 1/2) pathway upon *Salmonella* infection (Jones et al., 2008; Du and Galan, 2009). In addition, it was reported that AvrA has deubiquitinase activity and removes ubiquitin from IκB and β-catenin, two inhibitors of NF-κB, thereby preventing their degradation by the proteasome. The resulting increase of association with NF-κB attenuates the translocation of the transcription factor to the nucleus and attenuates the inflammatory response and cellular apoptosis (Ye et al., 2007). NF-κB functions as an important cellular hub of immune responses that is preferentially targeted by many effectors. Hence, *Salmonella* SseL is acting via a similar mechanism than AvrA (Le Negrate et al., 2008).

*E. coli* is producing a range of effectors with different functions and acting on different targets of the NF-κB signaling pathway. NleE displays methyltransferase activity and methylates TAB2/3, which in turn inhibits TRAF6-induced activation of the NF-κB pathway (Nadler et al., 2010; Newton et al., 2010; Vossenkamper et al., 2010). NleC is a zinc metallo-protease that degrades the NF-κB p65 subunit, thus blocking the induction of IL-8 (Yen et al., 2010). Another effector, NleH, blocks translocation of NF-κB into the nucleus without affecting IkBα degradation (Gao et al., 2009; Pham et al., 2012). NleD is a zinc-dependent metallo-protease specifically targeting the MAP kinases JNK and p38 but not Erk1/2, thereby blocking the nuclear translocation of the transcription factor AP-1 (Baruch et al., 2011).

Like *Salmonella* and *E. coli*, typical plant pathogenic bacteria such as *Pseudomonas spp.* or *Xanthomonas spp.* produce effectors that are translocated into the host cell via the T3SS. Genetic and functional analyzes have shown that a large number of T3E suppress defense responses that are normally induced by T3SS-deficient bacteria or by flg22 (Gohre and Robatzek, 2008; Dou and Zhou, 2012). These T3E affect PAMP/MAMP-induced signaling at different steps, from the early recognition events at the plasma membrane involving the PAMP/MAMP receptor complex to the late defense responses associated with the *de novo* synthesis of anti-microbial metabolites and proteins or callose deposition for cell wall reinforcement. AvrPto from *Pseudomonas syringae* interacts with the kinase domain of FLS2 (and other PAMP/MAMP receptors) *in planta* (Xiang et al., 2008) and/or with its co-receptor BAK1 (Shan et al., 2008). This interaction causes the inhibition of the kinase activity that is essential for the initiation of the signaling cascade (Xing et al., 2007). Very recently, it was shown that the tyrosine phosphatase activity of HopAO1 affects early immune signaling triggered by the receptor-like kinase EF-Tu Receptor (EFR) – and potentially FLS2 -, which perceives the elf18 peptide derived from the bacterial elongation factor Tu (EF-Tu) (Macho et al., 2014). Another effector from *P. syringae*, AvrPtoB, is a E3 ubiquitin ligase that causes degradation of FLS2 (Gohre et al., 2008). BIK1 is targeted by effectors originated from different phytopathogenic bacteria. *P. syringae* AvrPphB is a cysteine protease that causes degradation of BIK1 and related receptor-like cytoplasmic kinases such as PBS1 and PBLs (Zhang et al., 2010) whereas *Xanthomonas campestris* AvrAC displays uridylyl-transferase activity to interfere with the activation of BIK1 upon FLS2-BAK1 association (Feng and Zhou, 2012). Many T3E were shown to suppress PAMP/MAMP-dependent signal transduction downstream of the PRR complex. HopAI1, an effector with phosphothreonine lyase activity inactivates MPK3, MPK6, and MPK4 by dephosphorylating them (Zhang et al., 2007). HopF2 inhibits PAMP/MAMP-induced signaling by targeting MKK5 via its ADP-ribosyltransferase activity (Wang et al., 2010). There are increasing evidence that the nuclear transcriptional and post-transcriptional machinery represent a key target of bacterial effectors for the suppression of immunity-associated genes. For example, *X. campestris* XopD blocks the activity of the transcription factor MYB30 resulting in the suppression of basal immune responses (Kim et al., 2008; Canonne et al., 2011).

## EVOLUTIONARY CONSERVED CELLULAR HUBS OF IMMUNE RESPONSES AS PUTATIVE TARGETS OF ENTEROBACTERIA TYPE THREE EFFECTORS

A large yeast two-hybrid interaction matrix analysis with effectors from *P. syringae* and the filamentous oomycete *Hyaloperonospora arabidopsisdis*, both pathogens of *Arabidopsis*, suggests that taxonomically distant microorganisms have independently evolved effectors targeting an overlapping subset of proteins that are central components of the plant immune network (Mukhtar et al., 2011). By extension, cellular hubs that are evolutionary conserved among kingdoms are potentially prime choice targets of effectors. So far, a few studies show experimental evidence in favor of enterobacteria T3E being functional in plant cells. Notably, Ustün et al. (2012) showed that SseF from *S. enterica* induces a hypersensitive response (HR)-like cell death when transiently expressed in *Nicotiana benthamiana* (Ustün et al., 2012). Importantly, the HR-like phenotype is dependent on the co-chaperone SGT1 (Suppressor of G2 allele of skp1) and the protein NDR1 (Non-race specific Disease Resistance 1), which are essential for effector-triggered immunity , a form of race-cultivar specific resistance that is mediated by the recognition of effectors (mostly T3E) from adapted phytopathogenic bacteria by cytoplasmic plant resistance (R) proteins of the CC-NB-LRR (coiled-coil – nucleotide binding – leucine rich repeat domain) type. The function of R proteins is to monitor changes occurring to cellular components that are modulated by effectors. In the case of SseF, no plant interactors have been reported yet, thus, the virulence function of SseF, especially its ability to interfere with PTI/MTI remains elusive. SGT1 is conserved in both mammals and plants and in another study, it was shown that SspH2, an E3 ubiquitin ligase, interacts and modifies the activity of SGT1 to subvert immunity (Bhavsar et al., 2013). Effector-mediated protein ubiquitination to trigger degradation seems to be a common strategy adopted by mammals and plant pathogens to re-program cellular functions. SspH2 and other *Salmonella* ubiquitin ligases, e.g., SspH1, SlrP, or SopA might very well work *in planta* and target components of the PTI/MTI signaling pathway, like AvrPtoB does at the level of the PRR complex.

The MAP kinase cascades represent an obvious target of T3E action. SpvC interacts with *Arabidopsis* MPK6 by blocking the flg22-dependent post-translational activation of MPK3/6 and consequently the induction of the expression of typical PAMP/MAMP-induced marker genes (A. Schikora, personal communication). Because of the functional similarity with HopAI1, it is assumable that SpvC inactivates the plant MAP kinases through its phosphothreonine lyase activity.

Enterobacteria produce many effectors that modulate the function of the Rho family GTPases. Also the ROP (Rho of plant) family of GTPases is playing a determinant role in plant defense to pathogen attack (Moeder et al., 2005; Chen et al., 2010; Hoefle et al., 2011). It will be a very exciting challenge to elucidate to what extent the interaction between SptP and the *Arabidopsis* Rac1 homolog AtROP2 (A. Schikora, personal communication) affects the biochemical and biological function of this protein.

Overall these research findings give rise to the concept that *Salmonella* has deployed an arsenal of effectors able to manipulate both animal and plant basal immune defenses, which would be requested for successful growth and host colonization.

## CONCLUDING REMARKS

Until very recently it was assumed that disease caused by fruits and vegetables contaminated by enteropathogenic bacteria was due to post-harvesting handling. However, reports indicating that *Salmonella* and *E. coli* are able to actively grow and replicate in soil-grown vegetables suggest that plants may provide a niche for these bacteria (Holden et al., 2009; Schikora et al., 2012). In the case of *Salmonella*, it is still controversial whether the bacterium is able to enter the plant cell and replicate in specialized structures (the *Salmonella*-containing vacuoles) as it does in human epithelial cells. To date, none of the common plant pathogenic Gram- bacteria (*Pseudmonads, Xanthomonads*) was reported to penetrate into the cell but rather grow in the apoplast in an epiphytic modus. The reason for that is the presence of the plant cell wall, which may prevent bacterial uptake by macropinocytosis. Also, it is not proven whether the T3SS from *Salmonella* is adapted for translocation of effectors into plant cells. A major function of the T3E is to manipulate PAMP/MAMP-triggered immunity. As the molecular blocks of the signaling cascades are conserved between mammals and plants, it is tempting to speculate that *Salmonella* and *E. coli* have evolved effectors targeting components of the immune signaling pathway that are conserved among plants and animals. The biological relevance of pathogenic enterobacteria T3E manipulating signaling components of the plant immune system needs, however, to be addressed. Besides this aspect, these effectors can represent useful molecular tools to study the function of interacting plant proteins in their natural genetic background. This also suggests to search for the potential of effectors from other human and animal pathogens to re-program cellular functions in plants.

## Conflict of Interest Statement

The authors declare that the research was conducted in the absence of any commercial or financial relationships that could be construed as a potential conflict of interest.
